# Supplementary material for: The Data Use Ontology to streamline responsible access to human biomedical datasets
Source: Cell Genom. 2021 Nov 10;1(2):100028. doi: 10.1016/j.xgen.2021.100028 (PMC8591903; doi:10.1016/j.xgen.2021.100028)
Supplement: Document S1. Example implementation in the European Genome-Phenome Archive [file mmc1.pdf]

Cell Genomics, Volume *1*

Supplemental information

The Data Use Ontology to streamline

responsible access to human biomedical datasets

Jonathan Lawson, Moran N. Cabili, Giselle Kerry, Tiffany Boughtwood, Adrian Thorogood, Pinar Alper, Sarion R. Bowers, Rebecca R. Boyles, Anthony J. Brookes, Matthew Brush, Tony Burdett, Hayley Clissold, Stacey Donnelly, Stephanie O.M. Dyke, Mallory A. Freeberg, Melissa A. Haendel, Chihiro Hata, Petr Holub, Francis Jeanson, Aina Jene, Minae Kawashima, Shuichi Kawashima, Melissa Konopko, Irene Kyomugisha, Haoyuan Li, Mikael Linden, Laura Lyman Rodriguez, Mizuki Morita, Nicola Mulder, Jean Muller, Satoshi Nagaie, Jamal Nasir, Soichi Ogishima, Vivian Ota Wang, Laura D. Paglione, Ravi N. Pandya, Helen Parkinson, Anthony A. Philippakis, Fabian Prasser, Jordi Rambla, Kathy Reinold, Gregory A. Rushton, Andrea Saltzman, Gary Saunders, Heidi J. Sofia, John D. Spalding, Morris A. Swertz, Ilia Tulchinsky, Esther J. van Enckevort, Susheel Varma, Craig Voisin, Natsuko Yamamoto, Chisato Yamasaki, Lyndon Zass, Jaime M. Guidry Auvil, Tommi H. Nyrönen, and Mélanie Courtot

Latest DUO release content

Table S1. DUO terms as of February 23rd 2021. Each term has a stable identifier (column 1, “ID”), an optional shorthand (column 2) that can be used for visualisation purposes as shown on Figure 4, a label (column 3) and a textual definition (column 4).

Example implementation in the European Genome-Phenome Archive

1. Addition of DUO term(s) to EGA datasets

DUO can be added to datasets at the EGA by two routes.

The first, and currently most commonly used method is for the submitter to choose appropriate DUO term(s) for their dataset(s) using the most up to date version of the ontology, always available from http://purl.obolibrary.org/obo/duo.owl. Once they have identified the most appropriate DUO term(s) for their dataset(s) they provide them to the EGA by emailing helpdesk on helpdesk@ega-archive.org. The helpdesk team then runs an in-house script that assigns the DUO term(s) to the dataset(s) which in turn propagates through to our website.

The second method is done by the submitter themselves if they are submitting programmatically to the EGA through the use of the XMLs by adding it to the policy XML. In this instance the data use attribute in the XML is used to store the desired DUO term(s) along with the version which references the ontology used from a given build.

e.g,

```
<DATA_USES>
   <DATA_USE ontology="DUO" code="0000042" version="23-02-2021"/>
</DATA_USES>
```

In some instances, DUO can be used in combination with a modifier to allow more detailed description of how the data may be used. For example, DUO:0000007 (disease specific research) should be used in combination with terms from MONDO to describe specifically which disease research the data can be used for e.g., DUO:0000007 MONDO:0000996 would mean that the data could only be used for research into prostate lymphoma. In order to submit this programmatically the user would use a modifier attribute within the XML e.g.,

```
<DATA_USE ontology="DUO" code="0000007" version="23-02-2021">
  <MODIFIER>
    <DB>MONDO</DB>
    <ID>0000996</ID>
  </MODIFIER>
```

2. Rendering of DUO term(s) on the EGA website

On the EGA website, users can search using DUO terms and retrieve datasets of interest. For example, https://ega-archive.org/datasets/EGAD00001007598 shows a dataset consented for disease specific research (DUO:0000007) where the disease is further specified by using the MONDO term MONDO:0004992 - cancer.
